# Theories of Diseases—Preliminary Remarks

**Published:** 1879-08

**Authors:** B. F. Grove

**Affiliations:** Baltimore


### THEORIES OF DISEASES—PRELIMINARY REMARKS.

By B. F. GROVE, M.D., Baltimore.

"*Quot homines, tot sententia,*" is an old and a true saying, as applicable to medicine as to any other department of learning I know of, and therefore, since there are so many probably guessing, it may not be out of the way for me to do a little of it also.

With the teleological theory of the phenomena of the universe came, and still obtain various and varied narrow notions as to the relations and affinities of these phenomena, and among the many who accept that theory, many of these phenomena, nay, the vast majority of all the modes and conditions of matter and force, are looked upon as entities in and of themselves, each standing apart as an isolated fact, separate and distinct, the result of a special and independent proximate causation. Aristotle probably laid the foundation for broader views, but it remained for Goethe, Oken, Lamarek, Darwin, Spencer, to develop that system of generalization, which has so thoroughly changed the methods of scientific investigation. Goethe's views upon the morphological relations of the parts of plants, of the vertebræ, cannot but have greatly hastened those following him in rising from the contracted and artificial system of Linnæus, to the broad and national one of Lindley—destined at some future time to become still broader—in rising from the special physiology of an Agassiz, to the general one of Hæckel. As long as men blindly looked upon consciousness as an existence *per se*, just so long were they incapable of properly studying it; so soon, however, as they saw in it but effect of underlying material conditions, so soon were they able to appreciate its true nature. Science, and I mean more particularly that of medicine, had been until recently too prone to adapt its facts to its theories instead of these to its facts; and, accepting as true, certain hypotheses, had arbitrarily scanned its facts but to adapt them to those hypotheses, declaring these to be true until proven untrue—than which no position could be more false and erroneous. Fallacious ideas of the immediate relations of certain phenomena crept in, a necessary consequence of a want of appreciation of their more general connection; and *post hoc, ergo propter hoc*, though denied as a rule, is tacitly accepted and acted upon. The most flagrant errors arise also from incorrect notions of what constitutes evidence; that which is a mere possibility, or at most a probability, being affirmed as absolute certainty, in so far as it is possible for man to be certain at all. For example: A man receives an injury upon the head—a blow; meningitis develops itself; the patient dies; the post-mortem discovers no turbercles; and, therefore, the meningitis, which had no marked anatomy, is decided to be simple and traumatic, and consequently death has been brought about indirectly by the blow. Such logic is inconsistent. Firstly, because it asserts that tubercles are a *sine qua non* of tubercular meningitis—so called—which is tantamount to asserting that turbercle is auto genetic, in that it ignores the cause, which, efficient in producing tubercle, must have been equally so in including the meningitis. The chances are, at any rate, even, that this intrinsic condition may advance to the production of meningitis with or without tubercle; and the converse. Moreover, admit that their presence is essential; then the fact that none were discovered proves nothing, except that they were not found; for, to say that they were not present because not found, is the same as asserting that the investigator would have found them had they been present; and this is equïvalent to nothing more nor less than an enunciation of infallibility. Secondly, and a fortiori, it is fallacious in that it absolutely denies the possibility of coincidence in time of any causes which lead to meningitis, at least any two of them; thereby implicitly asserting that it is impossible for a man struck upon the head to be attacked with meningitis, unless it results from said blow; which is obviously absurd. Moreover, the number of blows received upon the head, not producing fracture, serious contusion of soft parts, encephalo-hematoma, and not followed by meningtis, are indefinite in number, say infinite, as compared with those followed by it; and, therefore, the amount of probability that, in any given case, meningitis will follow such blow, will be as infinity or indefinity to unity against the fact. Granting, for the sake of argument, that the number of cases, capable of inducing meningitis, are twelve, then the probability, that a meningitis, consequent upon such blow as the above, had been produced by it, would be eleven to one against the fact; for the possibility of coincidence in time of two or more cases cannot be denied. According to Surgeon-General Barnes, in 7,739 cases of gunshot wounds of scalp the deaths were 1.07 per cent.—93, counting 63 from unknown causes among them—from meningitis and encephalitis (?); by the most liberal estimate this would indicate consecutive meningitis in but 2.14 per cent. of the whole—331 cases of contused and lacerated scalp, being all that could be found, occurring from falls, and blows other than by weapons, all recovered. Now, allowing that a man with simple meningetis has an even chance of his life, we may accept that one case of the disease occurred in this number, the patient recovering, and this would be but 0 3 per cent. of the whole; a most liberal allowance. In 8,958 cases of injury to head, produced by every species of instrument from a pea-shooter to solid shot and caisson wheel, the deaths were—from all causes 305 or 3.4 per cent. of the whole; none of these cases were complicated by fracture, and many of the deaths were from tetanus, gangrene, shock, erysipelas, &c. Admit, however, that two-thirds of them were from meningitis, then the per centage of its occurrence in these 8,958 cases of injury serious and slight could not be more than 4 7⅓ per cent.; and this is, no doubt, higher than it actually was. Among 5,825,480 cases of disease and injury of all kinds, the ratio of encephalitis (?) was 0.035 per cent.; of meningitis alone, 0.023 per cent. From the above figures it will be seen that the estimate of probabilities in the hypothetical case are too small, rather than the reverse; they have nothing, however, to do with the amount of attention to be paid to injury of the calvarium and its coverings, however slight or severe. Again, an individual suffers from osteo-necrosis more or less severe and intractable; his physician, who has observed similar sequences before, predicts ultimate death from morbus Brightii; and, should this actually take place, congratulates himself upon any theory he may hold in regard to the connection of the two diseases, and also upon his prognosis. Now osteo-necrosis and morbus Brightii are certainly so common—at least they are by no means rare—that it is beyond a doubt within the range of possibility and probability, that in any given case the one may be concomitant or sequent to the other without the slightest causative relation existing between them, the prognostication was therefore in error. If now the disease of Bright was extremely rare—it is certainly far from that—and had frequently been observed to follow caries and necrosis, whilst the probabilities in the above case would be destroyed, the possibilities would not. And here again the prognostician would be in error, because in asserting the casual relation of the necrosis, he affirms that causes, which might be operative and efficient in others, could not be so in his patient, simply because of the necrosis, which would be sheer absurdity e. g., alcohol, than which no more potent factor in disease is more widely distributed: in Baltimore there is one rum shop to every seventy-five beings, irrespective of age or sex. The predilection of the Irish for whiskey is well known; the ratio of Bright's disease among them extremely high. Take an example not so complex: the iris; it is observed to contract and expand, and this action is explained by muscles, circular and radiating, under the influence of a controlling nerve. This is supposed to settle the matter; it is a hypothesis, bold but weak—it may be true or it may not. A certain number of observers testify to it as explanatory of certain physiological and clinical facts; but, as they differ among themselves, and there is no particular reason for believing one more than another, there is no reason for believing any; and therefore it is perfectly safe to believe none, looking for the explanation elsewhere or for additional facts. Concede, however, that they do agree, that they are all men of the highest authority, then, even in such a case, the declaration would still have to be considered upon its own intrinsic merits, and not from their dictum. From erroneous and hasty deduction, come equally incorrect diagnosis and ideas of treatment. A man is attacked with a disease—no matter what—and from observation in a certain number of apparently similar cases, the physician is led to use certain remedies in the hope of meeting with the same favorable results as in the preceding. Now admitting, for the sake of argument, that these remedies *were* efficient in those cases, how does he reach the application of the rule to the particular case? Suppose a Frenchman were expecting to meet an American friend, whom he had not yet seen, and that having already seen one hundred Americans, he has been observant enough to notice that *all* had light hair and blue eyes. Would the color of his friend's eyes be influenced by that of the hundred? If the Frenchman were to guess, could he approximate within more than an even chance of the truth? But the physician would be in a worse fix than the Frenchman, for he could not be certain as to the natural history of the disorders; he could not say whether the vis medicatrix would or would not have been sufficient. Taking all the so-called non-organic diseases, it is well known that a large proportion of them tend to spontaneous recovery, or are said to. Given any case in this class, by what process can an estimate of the value of remedies used be arrived at? If it could not be determined before hand that it would not terminate in death, how, in case of recovery, is it possible to assert that this was the result of treatment and not spontaneous in itself? Mr. Lister has devised a certain antiseptic method, but it devolves upon the adherents of the plan to show that cases terminating favorably under it would *not cæteris paribus* have ended equally as well without it. Than opium there is no drug whose sensible effects are better known, still there are numerous exceptions directly the result of its action, or produced by adventitious conditions. Say there is but one exceptional case known; another may occur, and though the chances that it will not do so are as an indefinitely great number to unity, still the possibility will always be present; who can determine when?

Certain effects of arsenic are equally patent. An individual has taken some doses; puffiness about the face appears, there is more or less œdema followed by anasarca, irritation in the alimentary canal, vomiting, serious inflammation of nervous system, convulsions, paralysis, death. Would it be safe to attribute this to the arsenic? no post-mortem having been made, for the reason that the taking of the drug and the sequence of symptoms were facts susceptible of clearest proof. Obviously it would not; and moreover it would be equally unsafe, had the post-mortem given the first evidence that the poison had been taken, to attribute death to it, *merely* from the fact of its presence, and for the reason assigned above, that it would grant immunity to the individual from other causes, simply because one *capable*, under certain circumstances, of producing the same effects happened to be present.

The above arguments are not intended to touch medical deductions, as subjects of inquiry in themselves, but only as they are results of certain species of reasoning, since it is perfectly evident that fact or reality, owing its truth to nothing but its own inherent character, is objectively considered, quite independent of all methods of reasoning, though it is only by a correct method that it can certainly be attained. Had Neptune never been discovered, or had Le Verrier inferred its existence and orbit merely *because* Saturn was discovered beyond Jupiter, the reality of its existence would still have been the same; and this, notwithstanding the fact that such a result under the second supposition, would be logically absurd, if not preposterous; neither could it be confirmed by acquiescence nor destroyed by negation. Nor are they for the purpose of destroying belief, but simply for that of creating doubt; since, as faith and belief are the elements of stagnation, so is doubt that of progress, and though this is strictly true, by that very fact is doubt the very essence of scientific conviction. It is obscure belief, founded upon illogical deduction, that causes so much disagreement among physicians; some of whom imagine they avoid the difficulty by adopting that thoroughly imaginary and ideal point which they call "the happy mean." It is the same which causes the *vulgus* to pass from "allopath" to homœopath, and from both to eclectic; not perceiving that but *one* alone can be true, and that this once admitted all others must be as utterly false as this is true. I propose, therefore, in this paper to enquire into certain theories of disease and their treatment; and to find out, if I can, how much of that superstition there is in medicine, which believes hydrophobia or "dog-days" as existing and possible only in summer; and which, from an obscure and misunderstood word, can create a nation of "beef-eaters."—*Maryland Medical Journal.*

